# CDC’s COVID-19 International Vaccine Implementation and Evaluation Program and Lessons from Earlier Vaccine Introductions

**DOI:** 10.3201/eid2813.212123

**Published:** 2022-12

**Authors:** Heidi M. Soeters, Reena H. Doshi, Monica Fleming, Oluwasegun Joel Adegoke, Uzoamaka Ajene, Brooke Noel Aksnes, Sarah Bennett, Erin F. Blau, Julie Garon Carlton, Sara Clements, Laura Conklin, Melissa Dahlke, Lindsey M. Duca, Leora R. Feldstein, Jane F. Gidudu, Gavin Grant, Margaret Hercules, Ledor S. Igboh, Atsuyoshi Ishizumi, Sara Jacenko, Yinka Kerr, Nuadum M. Konne, Shibani Kulkarni, Archana Kumar, Kathryn E. Lafond, Eugene Lam, Ashley T. Longley, Margaret McCarron, Apophia Namageyo-Funa, Nancy Ortiz, Jaymin C. Patel, Robert T. Perry, Dimitri Prybylski, Prianca Reddi, Omar Salman, Courtney N. Sciarratta, Talya Shragai, Akshita Siddula, Ester Sikare, Dieula Delissaint Tchoualeu, Denise Traicoff, Alexandra Tuttle, Kerton R. Victory, Aaron Wallace, Kirsten Ward, Man Kai Alyssa Wong, Weigong Zhou, W. William Schluter, David L. Fitter, Anthony Mounts, Joseph S. Bresee, Terri B. Hyde

**Affiliations:** Centers for Disease Control and Prevention, Atlanta, Georgia, USA

**Keywords:** COVID-19, SARS-CoV-2, severe acute respiratory syndrome coronavirus 2, viruses, respiratory infections, zoonoses, vaccines, implementation, evaluation, coronavirus disease, Ebola, influenza, meningococcus

## Abstract

The US Centers for Disease Control and Prevention (CDC) supports international partners in introducing vaccines, including those against SARS-CoV-2 virus. CDC contributes to the development of global technical tools, guidance, and policy for COVID-19 vaccination and has established its COVID-19 International Vaccine Implementation and Evaluation (CIVIE) program. CIVIE supports ministries of health and their partner organizations in developing or strengthening their national capacities for the planning, implementation, and evaluation of COVID-19 vaccination programs. CIVIE’s 7 priority areas for country-specific technical assistance are vaccine policy development, program planning, vaccine confidence and demand, data management and use, workforce development, vaccine safety, and evaluation. We discuss CDC’s work on global COVID-19 vaccine implementation, including priorities, challenges, opportunities, and applicable lessons learned from prior experiences with Ebola, influenza, and meningococcal serogroup A conjugate vaccine introductions.

In March 2020, the World Health Organization (WHO) characterized COVID-19 as a global pandemic, driving a race to develop vaccines against SARS-CoV-2, the virus that causes COVID-19. Nine months later, the first COVID-19 vaccine was approved for widespread use in the United Kingdom; the vaccination program there launched on December 8, 2020 (*1*). In rapid succession, the United States issued an emergency use authorization for, recommended, and began administration of COVID-19 vaccines as well (*2*), and WHO issued the first emergency use listing (EUL) and policy recommendations for COVID-19 vaccines (*3*). As of April 2022, >11 billion doses of COVID-19 vaccines have been administered worldwide (*4*), and a total of 10 COVID-19 vaccines have been issued under EUL from WHO (*5*).

The Access to COVID-19 Tools (ACT) Accelerator is the coordinated global effort to develop diagnostic, treatment, and prevention tools to fight COVID-19 (*6*). COVID-19 Vaccines Global Access (COVAX) is the vaccines pillar of the ACT Accelerator and aims to accelerate development and manufacture of COVID-19 vaccines and to guarantee fair and equitable access for every country in the world (*7*). WHO; the Coalition for Epidemic Preparedness Innovations (CEPI); and Gavi, the Vaccine Alliance, co-lead COVAX. As of April 2022, a total of 145 countries were participating in COVAX (*8*), including both funded and self-financing economies (*9*). The US government is the largest contributor to COVAX and has committed US $4 billion in funding (*10*) and committed to donating >1.1 billion vaccine doses (*11*) as of October 2021; the Centers for Disease Control and Prevention (CDC) contributes assistance to COVAX as a key technical partner. Although COVAX is the single largest mechanism for COVID-19 vaccine procurement globally, countries may also gain access to doses via national production, bilateral agreements with vaccine manufacturers, or bilateral donations.

CDC supports international COVID-19 vaccination efforts and COVAX by participating in global-level technical working groups, collaborating with global immunization partners to create tools and guidance, and gathering and synthesizing evidence to support new policy and global guidance. CDC also supports global COVID-19 vaccine implementation as a key component of CDC’s Strategy for the Global Response to COVID-19 (*12*). CDC anticipates these activities will reduce the COVID-19 burden in partner countries while strengthening partner countries’ capacities to vaccinate their populations against future vaccine-preventable diseases that pose an epidemic or pandemic threat.

To support ministries of health in developing or strengthening their national capacities for the planning, implementation, and evaluation of COVID-19 vaccination programs, CDC established the COVID-19 International Vaccine Implementation and Evaluation (CIVIE) program. We describe the CIVIE program; challenges and opportunities with global COVID-19 vaccine implementation; and applicable lessons learned from prior experiences with Ebola, influenza, and meningococcal serogroup A conjugate vaccine introductions.

## CIVIE

**Figure 1:**
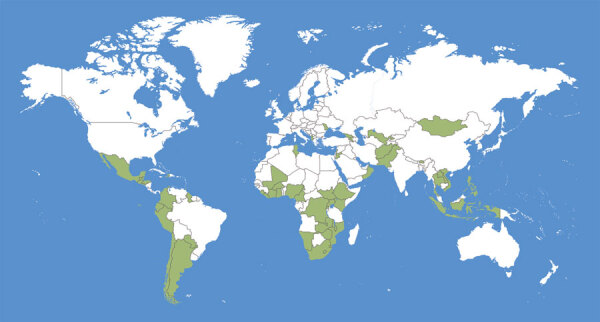
Green shading indicates the 55 countries supported by the Centers for Disease Control and Prevention’s COVID-19 International Vaccine Implementation and Evaluation program in fiscal year 2021.

CDC established the CIVIE program in 2020 to help country ministries of health and their partner organizations effectively introduce, deploy, manage, and evaluate COVID-19 vaccines, with the additional goal of establishing sustainable programs for the delivery of immunizations throughout the life-course (*13*). CIVIE initially prioritized specific low- and middle-income countries (LMICs) for potential CDC support for COVID-19 vaccine implementation. CIVIE evaluated each country by factors including level of interest, the presence of a CDC office or staff in that country, the existence of CDC-supported programs, and eligibility to receive donor-funded COVID-19 vaccines through COVAX (Figure 1).

CIVIE supports countries in implementing their national deployment and vaccination plans for COVID-19 vaccines (*14*) by working with the countries’ ministries of health to identify specific activities that would benefit from CDC technical or financial support. In countries with in-country CDC staff, CIVIE primarily works with ministries of health through CDC staff; in countries without in-country CDC presence, CIVIE either engages directly with ministries of health or supports them via regional CDC offices, CDC-funded implementing partners, or WHO offices at the country or regional level.

**Figure 2:**
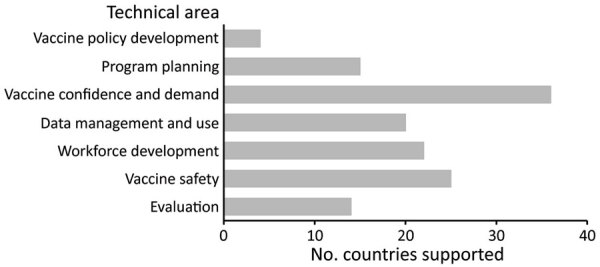
Technical areas of support provided to countries by the Centers for Disease Control and Prevention’s COVID-19 International Vaccine Implementation and Evaluation (CIVIE) program in fiscal year 2021

CIVIE’s 7 priority areas for country technical assistance are vaccine policy development, program planning, vaccine confidence and demand, data management and use, workforce development, vaccine safety, and evaluation (Table 1). CIVIE chose these technical areas to leverage CDC’s technical expertise and comparative advantages for supporting country-level vaccine implementation and evaluation (*15*), on the basis of lessons learned from other vaccine introductions. In fiscal year 2021, CIVIE supported 55 countries, representing 27% of the world’s population (Figure 1); vaccine confidence and demand and vaccine safety were the most commonly requested areas for CDC country support (Figure 2).

**Table 1:** CDC priority technical areas to support global COVID-19 vaccine implementation through the COVID-19 International Vaccine Implementation and Evaluation (CIVIE) program

Technical area	Examples of CDC-supported activities
Vaccine policy development	Assist with data review to inform prioritization and planning for vaccination of risk groups
	Support and strengthen national-level decision making and National Immunization Technical Advisory Groups via trainings and workshops
Program planning	Support microplanning for populations targeted for vaccination
	Help design logistical and distribution plans for different vaccination scenarios or products
Vaccine confidence and demand	Develop and provide standard tools for country-level adaptation to collect data on behavioral and social barriers to vaccine uptake
	Provide support to assess and manage the effect of infodemics* on vaccine confidence and uptake
	Provide messaging and communications technical assistance, materials, and tools
Data management and use	Provide technical assistance to rapidly assess, develop, implement, and monitor data management systems and tools used for COVID-19 vaccine introduction and safety monitoring
Workforce development	Conduct rapid performance assessments to understand workforce-related barriers and facilitators to delivering COVID-19 vaccine
	Provide evidence-based tools and techniques for improving supervision
Vaccine safety	Strengthen passive or enhanced surveillance for adverse events following immunization
	Use active surveillance or special studies to address key questions on COVID-19 vaccine safety
	Ensure preparedness to respond to safety events through vaccine-related event response planning
Evaluation	Support post-introduction evaluations using standard WHO tools
	Conduct targeted evaluations of vaccine effectiveness to address key global evidence gaps

*Infodemic, “overabundance of information during a disease outbreak” (*17*).

To carry out this work, CDC received funding from both the Coronavirus Aid, Relief, and Economic Security Act in 2020 and the American Rescue Plan in 2021. CDC has awarded funds to the Task Force for Global Health (TFGH) as a main implementing partner; TFGH then subawards funding to in-country partners. In addition, CIVIE has provided funding to the WHO headquarters and regional offices to indirectly support global, regional, and country-level COVID-19 vaccine implementation. CDC’s support for COVID-19 vaccine implementation and evaluation in fiscal year 2021 was coordinated with support provided to countries from other US government entities, such as US Agency for International Development (USAID) (*10*) and the US President’s Emergency Plan for AIDS Relief (PEPFAR), as well as from multilateral partners such as WHO (*7*), Gavi (*8*), UNICEF (*16*), and other global partners. Specific examples of CDC’s coordination with partners and technical activities in countries to accelerate progress toward widespread and equitable access to safe and effective COVID-19 vaccines have been published (*17*,*18*).

## Challenges

The COVID-19 vaccine rollout faced many challenges. Although COVID-19 vaccines were rapidly developed and manufactured, getting the vaccines delivered to countries, distributed within countries, and administered worldwide is a complex interdependent effort (*7*). Challenges we observed during the initial rollout were insufficient manufacturing capacity, supply constraints, the overwhelming and simultaneous demand for vaccination, inequitable vaccine distribution and access, partner coordination challenges, a complicated and evolving vaccine product landscape, multidose schedules, a limited evidence base for some vaccine products, staffing shortages, and overburdened healthcare workers. In addition, many countries were inadequately prepared to monitor vaccine safety and address public concerns about COVID-19 vaccines, and an overabundance of information, including misinformation (*19*), contributes to low vaccine confidence in many populations.

Unlike childhood vaccination programs, which are present in all countries, 38% of countries lacked adult vaccination programs in 2018 (*20*), and specific immunization programs for healthcare workers are not present in many countries. The COVID-19 vaccine rollout began by targeting healthcare workers, older adults, and other special populations who are most at risk for severe disease or death from COVID-19, followed by other members of the general adult population (*21*). This process required devising and communicating new strategies for vaccine implementation that were different from the typical routine childhood immunization programs. Furthermore, these new strategies had to be tailored to individual countries and target populations for vaccination, which required continuous evaluation and adaptive communication strategies.

## Opportunities

Despite these challenges, the global introduction of COVID-19 vaccine presented opportunities for improving and modernizing immunization programs. Vaccination provides a path out of the COVID-19 pandemic; the world’s current focus on immunization can be leveraged to ensure a successful COVID-19 vaccine rollout and strengthen the demand for and confidence in vaccination against all vaccine-preventable diseases. Because the COVID-19 vaccines were made available for adult populations first and were subsequently approved for younger age groups, COVID-19 vaccination provides an excellent opportunity to emphasize the life-course approach to vaccination (*22*), a key element of the WHO Immunization Agenda 2030 (*13*).

CIVIE’s financial and technical support to ministries of health for rapid implementation and evaluation of COVID-19 vaccines offers many benefits, including the strengthening of existing partnerships and the formation of new collaborations beyond the traditional immunization partner organizations. With continued support, the resulting evidence-based improvements in immunization systems could lead to long-term benefits such as the establishment of new adult and healthcare worker vaccination platforms, strengthened national immunization programs, introductions of non–COVID-19 vaccines that were placed on hold during the pandemic, and improved country readiness for future vaccine introductions, including those in response to public health emergencies.

Because the CIVIE program was created very early in the COVID-19 vaccine introduction process, it provided a foundation upon which new activities could be launched as vaccine rollout progressed. In the second year of the program, CIVIE continued to respond to the evolving needs of global COVID-19 vaccination implementation, such as increased vaccine supply and the need to address growing inequities in vaccination coverage, by continuing system-strengthening activities and further expanding support in the areas of postintroduction evaluations, vaccine effectiveness studies, vaccine campaigns or high-throughput vaccination planning and implementation, and vaccination in humanitarian settings.

## Lessons Learned from Previous Vaccine Introductions

Despite the unprecedented nature of the COVID-19 pandemic and the resulting global COVID-19 vaccination effort, the CIVIE program found applicable lessons in previous introductions of Ebola, influenza, and meningococcal serogroup A vaccines (Table 2). Although many lessons, both positive and negative, have been learned through experiences with prior vaccine introductions, we have chosen to focus on these particular vaccines given the CIVIE team’s collective background experience. We selected illustrative examples that informed the strategy for COVID-19 vaccine implementation and high-level lessons learned, although certainly county-level and subnational lessons have been learned as well.

**Table 2:** Applicable lessons for global COVID-19 vaccine implementation learned from prior vaccine introductions

Vaccine	Lessons learned
Ebola	Experience with vaccine prioritization in the setting of vaccine supply constraints during an outbreak
	How to identify and vaccinate healthcare workers
	The importance of strong community engagement to build trust and vaccine confidence
Influenza	National capacities in microplanning, accessing target vaccination groups, workforce training, and conducting vaccination campaigns strengthened via seasonal influenza programs
	The Partnership for Influenza Vaccine Introduction program provided a model structure that formed the basis for the COVID-19 International Vaccine Implementation and Evaluation program
Meningococcal serogroup A	Experience with rapid mass vaccination campaigns for adults in low-resource settings
	The importance of clear communication to the public
	Methods for ensuring vaccination program sustainability

### Ebola Vaccine

Experience with the Ebola vaccine has highlighted some of the difficulties associated with the introduction of new vaccines during public health emergencies (e.g., vaccine supply constraints, identification and vaccination of healthcare workers, and the importance of strong community engagement to build trust and vaccine confidence). During the large West Africa Ebola virus disease (EVD) outbreak in 2014–2016, Ebola vaccine development was expedited, driven by the gravity of the public health emergency and the need for rapid access to a safe and effective vaccine against Ebola viruses (*23*–*26*). Since then, >300,000 persons have been vaccinated with rVSVΔG-ZEBOV-GP (ERVEBO; Merck & Co., Inc., https://www.merck.com) during multiple EVD outbreaks in the Democratic Republic of the Congo (DRC), Guinea, Uganda, South Sudan, Burundi, and Rwanda, using a vaccination strategy targeting EVD case contacts, contacts of contacts, healthcare workers, and frontline workers (*27**–**29*). A second Ebola vaccine option, the 2-part regimen of Ad26.ZEBOV (Zabdeno; Janssen, https://www.janssen.com) and MVA-BN-Filo (Mvabea; Janssen), is now recommended as preventive vaccination for at-risk persons, such as healthcare workers and frontline workers in neighboring countries where EVD outbreaks may spread (*30*).

As we have seen with COVID-19 vaccines, supply constraints have limited the use of the Ebola vaccine during outbreaks. Limited quantities meant that vaccination strategies had to be tailored based on the vaccine and disease characteristics, risk-benefit analyses for different target populations, and country-specific contexts. WHO developed the Strategic Advisory Group of Experts on Immunization (SAGE) roadmap for prioritizing use of COVID-19 vaccines in the context of limited supply as a tool for countries to optimize the benefits from COVID-19 vaccines, based on public health goals, vaccine access, and various vaccination coverage scenarios (*21*). Ebola vaccination strategies have similarly prioritized most-at-risk populations, such as healthcare workers. However, preventive Ebola vaccination activities in Uganda, Rwanda, South Sudan, Burundi, Sierra Leone, and Liberia highlighted the challenges associated with quickly defining, identifying, and vaccinating healthcare worker populations. These challenges included unknown population estimates; high turnover of facility-based healthcare workers, which limits knowledge accumulation and makes it difficult to maintain high vaccination coverage; and the fact that EVD outbreaks often occur in rural areas where traditional healers and community health workers are more difficult to identify (*31*). These challenges necessitated strong microplanning and developing a healthcare worker registry to ensure accurate estimates of vaccine doses (*31*), both which are applicable to COVID-19 vaccination efforts. The Ebola vaccine experience also presaged the need to rapidly develop and distribute locally appropriate job aids and just-in-time training, as access and acceptance of technology continue to increase; similarly, workforce development is a strong area of focus for COVID-19 vaccine.

The experience with the Ebola vaccine has reinforced the crucial role of social and behavioral science in immunization programs, generating many lessons learned about the importance of communication and strong community engagement to build vaccine trust (*32*–*34*). Of note, Ebola vaccine prioritization efforts led to confusion and mistrust in the community because of concerns about vaccine equity, thereby undercutting vaccine confidence (*35*,*36*). In addition, rumors about Ebola vaccine eligibility and safety circulated on both traditional and social media, which likely reduced vaccine uptake (*34*–*36*). In DRC, rapid surveys were conducted to monitor community perceptions, vaccine acceptance, and misinformation; in addition, local partners regularly compiled community feedback from focus groups and key informant interviews to inform response interventions and improve vaccination uptake (*35*,*36*). Similar strategies for understanding community perceptions of COVID-19 vaccines (e.g., knowledge, attitudes, and practices surveys; health communication; and social listening activities) have been a key part of CIVIE’s support to partner countries and have been used to develop culturally appropriate materials that convey accurate information and improve local COVID-19 vaccine uptake.

### Influenza Vaccine

Seasonal influenza vaccines have been used in immunization programs in high-income countries for decades but remain underused in LMICs. For example, in 2017, countries in the African, Eastern Mediterranean, and South-East Asian WHO regions represented 49% of the global population but received 6% of all manufactured doses of influenza vaccine (*37*). The low uptake of influenza vaccines globally results in a substantial annual preventable disease burden and missed opportunities to strengthen pandemic vaccine preparedness through the annual planning and deployment of influenza vaccines. Seasonal influenza vaccination provides countries annual opportunities to strengthen capacity in microplanning, accessing target groups likely to be included in early pandemic vaccination priorities (e.g., healthcare workers, older adults, pregnant persons), training workforces, and conducting time-limited campaigns. A review of the 2009 influenza A(H1N1) pandemic vaccine deployment found that countries with existing seasonal influenza programs at the onset of the pandemic were able to deploy pandemic vaccines more quickly than those without such programs (*38*). Similar regional reviews confirmed that successful H1N1 vaccination in 2009 required capacities that were built or strengthened through seasonal influenza vaccination (*39*).

To support expanded influenza vaccination, CDC initiated the Partnership for Influenza Vaccine Introduction (PIVI) in 2013 with the TFGH, and in coordination with WHO (*40*). PIVI has supported LMICs to plan, implement, and evaluate influenza vaccination programs by providing access to influenza vaccine doses and targeted technical assistance. PIVI partner countries have reported that invaluable capabilities were developed as part of their influenza programs (e.g., policy development, microplanning, communications, and health worker training), which in turn accelerated the deployment of COVID-19 vaccines. In addition, the PIVI model of bilateral engagements with ministries of health to provide funding and technical assistance has formed the basis for CIVIE’s country engagement approach, factored into the initial country prioritization, and enabled rapid provision of assistance. Building on these direct engagements with LMICs, CIVIE is working with WHO, TFGH, and other partners to evaluate whether the presence of seasonal influenza vaccination programs or other adult vaccination programs is associated with more successful national COVID-19 vaccination programs. If the presence of influenza vaccination programs improves national pandemic responses, that evidence strengthens the argument for continued and increased investment in adult and healthcare worker vaccination programs.

### Meningococcal Serogroup A Conjugate Vaccine

Meningococcal serogroup A conjugate vaccine (MACV), MenAfriVac, was developed to prevent the predominant cause of meningitis epidemics in the Africa meningitis belt. Starting in 2010, MACV was implemented via mass vaccination campaigns targeting persons 1–29 years of age (*41*). MACV was the earliest known new vaccine to be initially introduced in the WHO Africa region via mass vaccination campaigns instead of routine childhood immunization (*41*); MACV was later integrated into national childhood immunization programs to ensure continued community protection. Some key lessons learned from MACV rollout that help inform global COVID-19 vaccination efforts included how to launch rapid mass vaccination campaigns for adult populations in low-resource settings, the importance of clear communication to the public, and how to ensure vaccination program sustainability. 

Conducted in 24 of 26 meningitis-belt countries to date (*42*), MACV mass campaigns were immensely successful. MACV was met with extremely high community acceptance (*43*), evidenced by 98% administrative coverage among the target populations (*41*), and resulted in a near disappearance of *Neisseria meningitidis* serogroup A meningitis from the region (*44*). Some elements that contributed to the success of these mass campaigns that are also crucial components of COVID-19 vaccine rollout were strong global coordination, country engagement, early and adequate microplanning, cascade training, community engagement, deployment of technical assistance staff, intensive supportive supervision, and adequate provision of vaccines and logistics (*41*,*43*).

Another factor contributing to the success of MACV mass campaigns was clear communication from the governments and partners about the risks of the disease versus the benefits of vaccination. Many of the communities that were offered vaccination with MACV had long collective experience with meningitis and personally knew those who had had the disease, which has a high case-fatality ratio. The collective fear of meningitis made the benefits of vaccination clear, and persons were very willing to seek MACV for themselves and their children. Although COVID-19 has proven itself to be a deadly disease, with >6 million deaths reported worldwide by April 2022 (*4*), it does not have the same severity or case-fatality ratio as meningitis or EVD. Perceptions about the risk of COVID-19 compared with the benefits of COVID-19 vaccination differ (*45*) and can be influenced by misinformation; this variability in risk perception necessitates tailored communication strategies.

Finally, the experience with MACV offered lessons learned regarding vaccination program sustainability. Although MACV was rolled out via mass vaccination campaigns initially, WHO recommends that MACV be introduced into the childhood immunization program for children 9–18 months of age after the completion of a country’s mass campaign to sustain population-level immunity, (*46*). However, this next step of childhood immunization has proceeded slowly; only 15 of the 24 meningitis belt countries that conducted mass campaigns have followed through with MACV introduction for children (*47*). This delay may in part be a result of the success of the mass campaigns, which dramatically decreased the disease burden and may thereby have reduced the perceived urgency for MACV introduction for children (*41*). Although the continued need for COVID-19 vaccination beyond this pandemic phase is unclear, the potential need for ongoing booster doses would require countries to develop sustainable ways to integrate COVID-19 vaccination into their national immunization programs for children, adolescents, and adults.

## Conclusion

Although the global COVID-19 vaccine rollout is an unprecedented response to a major global pandemic and is faced with many and ever-changing challenges, applicable lessons have been and can be learned from experience with other vaccine introductions. CIVIE’s support to countries builds on lessons learned from other global vaccine initiatives to help LMICs deploy and evaluate COVID-19 vaccines, thereby reducing disease burden and transmission in their countries while also reducing the threat of COVID-19 globally. These activities can help expand sustainable programs for the delivery of immunizations throughout the life-course while strengthening partner countries’ capacities to vaccinate their populations against current or future vaccine-preventable diseases.
